# Sialylation in Thyroid Carcinoma: An Overview of Mechanisms, Markers, and Therapeutic Opportunities

**DOI:** 10.7150/jca.122078

**Published:** 2026-01-01

**Authors:** Chengyuan Li, Jianing Zhou, Lijun Zhang, Yan Si, Xiang Zhang, Meiping Shen

**Affiliations:** Department of General Surgery, The First Affiliated Hospital of Nanjing Medical University, No. 300 Guangzhou Road, Nanjing, 210029, China.

**Keywords:** thyroid carcinoma, sialylation, sialic acid-binding immunoglobulin-like lectins

## Abstract

Thyroid carcinoma (TC) is the most prevalent malignancy of the endocrine system, with its incidence rising annually worldwide. Post-translational modifications and epigenetic changes have been documented to be pivotal in the initiation, progression, and malignant transformation of TC. In addition to mediating biological processes such as cell recognition, signal transduction, and immune regulation, these modifications can also significantly impact the development and metastasis of various cancers. Among these, sialylation is identified as a key post-translational modification, showing close associations with the invasiveness and metastatic potential of TC. This review aims to provide an overview of the current understanding of sialylation in TC, highlighting its underlying mechanisms and examining its roles in cell proliferation, invasion, and immune evasion. Additionally, this study intends to explore the potential of targeting sialylation as a novel therapeutic approach, providing new perspectives for TC prevention and treatment, as well as the development of new therapeutic agents.

## 1. Introduction

Thyroid carcinoma (TC) is the most prevalent malignancy of the endocrine system, ranking as the seventh most common cancer worldwide given its newly diagnosed cases of over 821,000 in 2022^1^. Its development and progression may be attributed to various risk factors, such as chromosomal and genetic mutations, iodine intake, radiation history, autoimmune thyroid diseases, gender, estrogen, obesity, lifestyle changes, and environmental pollutants, etc^2^. Based on tumor origin and differentiation status, TC is classified into several subtypes, including papillary TC (PTC), follicular TC (FTC), poorly differentiated TC (PDTC), anaplastic TC (ATC), and medullary TC (MTC), the latter of which originates from parafollicular C cells. Among these, PTC is the most common histotype, accounting for approximately 90% of all TCs. PTC and FTC are collectively referred to as differentiated TC (DTC)^3^.

The occurrence and progression of TC involve complex molecular mechanisms and regulatory networks. Protein modifications serve as crucial regulatory mechanisms in various physiological and pathological processes intracellularly, which have been highly concerned in recent decades. These modifications include phosphorylation, glycosylation, ubiquitination, methylation, etc^4^. Beyond impacts on the structure, function, and stability of proteins, such modifications can also regulate intracellular signaling, immune response, and biological processes such as cell proliferation, migration, and apoptosis^5^,^6^. Advancement in research within this field has highlighted the critical role of abnormal protein modifications in tumor initiation, progression, and the acquisition of malignant traits, offering new insights into the mechanisms underlying TC and potential therapeutic strategies. Among these modifications, glycosylation has gained increasing interest in oncology. It refers to a specific enzymatic process in which carbohydrate moieties are added to specific amino acid residues on proteins by glycosyltransferases, forming glycosidic bonds. It encompasses various modifications, such as sialylation, fucosylation, and O-glycan truncation^7^. Abnormal sialylation is one of the most common glycosylation progresses in cancer, and has become a major area of focus in tumor research^8^-^10^. Existing evidence indicates the important position sialylation occupied in the development of TC. Accordingly, this review aims to summarize the specific roles of sialylation in TC.

## 2. Sialic Acids and Sialylation

### 2.1 Biochemical Characteristics of Sialic Acids

Sialic acids are a class of acidic sugar molecules containing nine carbon atoms, with wide distribution on the surface of animal and plant cells, as well as on certain biomolecules. Typically, sialic acids locate at the ends of glycoproteins or glycolipids, where they serve as 'bridge' molecules that facilitate cell-cell and cell-extracellular matrix interactions^11^. These compounds were first isolated by Gunnar Blix in 1936 from submandibular gland mucoproteins, named 'sialic acid'^12^. In the early 1940s, Ernst Klenk isolated acidic gangliosides composed of neurotransmitters, fatty acids, hexoses, and neuraminic acids abundant in the brain^13^. In 1957, Blix and colleagues found that neuraminic acids shared structural similarities with sialic acids^14^. Sialic acids exhibit distinctive structures, characterized by a carbon atom bearing both an amine and a carboxyl group, imparting strong acidity and hydrophilicity. The most common forms of sialic acids are N-Acetylneuraminic acid (Neu5Ac or ManNAc) and its derivative, N-Glycolylneuraminic acid^15^. In malignant tumors, aberrant sialylation of cell surface glycans may alter charge properties and steric hindrance, thereby facilitating tumor cell dissociation, invasion, and metastasis directly^16^,^17^.

### 2.2 Enzymatic Process of Sialylation

**Figure 1:**
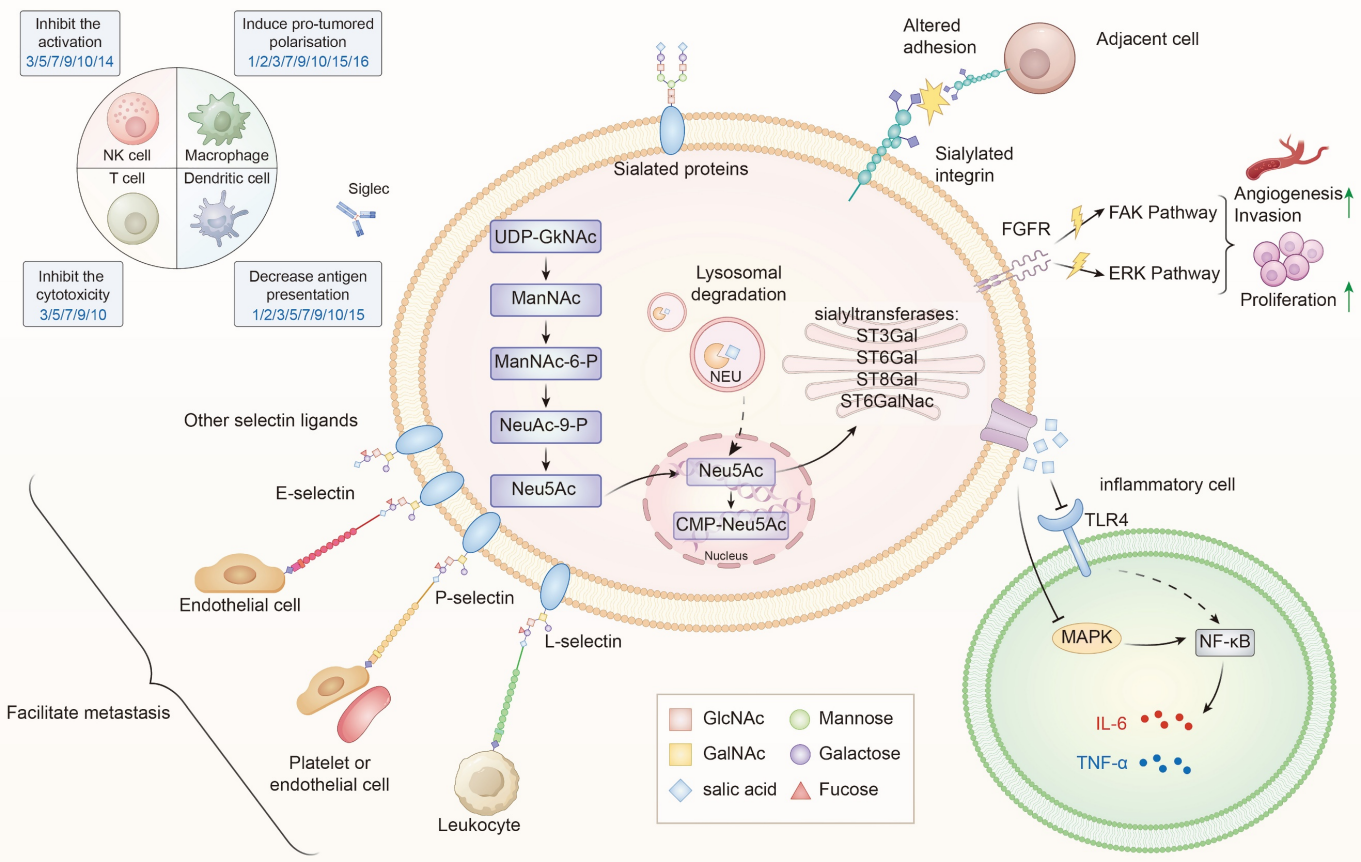
** The role of sialylation in thyroid cancer progression.** Sialylation in TC is regulated by STs (ST3Gal, ST6Gal, ST8Gal ang ST6GalNAc) and NEUs, which dynamically control the addition or removal of sialic acids. Within the cell, sialic acids are synthesized from UDP-GlcNAc through the ManNAc-Neu5Ac pathway, subsequently activated to CMP-Neu5Ac in the nucleus and utilized in the Golgi apparatus for glycoprotein and glycolipid sialylation^83^. Aberrant sialylation leads to multiple oncogenic effects: (1) Altered adhesion and metastasis—sialylated integrins and selectin ligands (E-, P-, L-selectins) facilitate interactions with endothelial cells, leukocytes, and platelets, promoting tumor dissemination; (2) Activation of signaling pathways—sialylated FGFR and integrins activate FAK and ERK cascades, driving proliferation, invasion, and angiogenesis; (3) Immune evasion—sialylated ligands engage Siglec receptors on NK cells, T cells, dendritic cells, and macrophages, inhibiting cytotoxicity, dampening activation, and inducing pro-tumoral polarization; (4) Inflammatory modulation—via TLR4-MAPK-NF-κB signaling, sialylation influences cytokine secretion (IL-6, TNF-α) and shapes the tumor microenvironment. Collectively, these processes contribute to the malignant progression of TC by promoting proliferation, invasion, immune suppression, and metastatic spread^16^,^84^.

Sialylation, or the covalent addition of sialic acids to the terminal end of glycoproteins, is a biologically significant modification involved in processes such as embryonic development, neurodevelopment, reprogramming, tumorigenesis, and immune responses^9^,^18^-^20^. Over 50 subtypes of sialic acids have been identified, with Neu5Ac being the most prevalent form in humans^21^.​ Figure 1 provides an overview of the sialylation process.

## 3. Role of Sialylation in Tumorigenesis

In TC, abnormal sialylation has been directly implicated in tumor initiation and progression. Early studies demonstrated that elevated serum and tissue levels of total sialic acids (TSA) could serve as markers for TC^22^. More recent investigations revealed that dysregulated expression of sialyltransferases (STs), such as ST6GAL1 and ST6GAL2, was associated with lymph node metastasis, advanced clinical stage, and poor prognosis in TC patients^23^,^24^. These findings underscore the clinical relevance of sialylation in TC.

Beyond TC, evidence from other malignancies provides additional mechanistic insights. Abnormal sialylation, mainly regulated by STs and neuraminidases (NEUs), can disturb the balance of sialic acids and drive uncontrolled proliferation, invasion, migration, and immune suppression^25^-^27^. To date, existing research has identified 20 STs and 4 NEUs, which can mediate α-2,3, α-2,6, or α-2,8 linkages^28^. Table 1 summarizes the specific roles of related enzymes in TC, while Table 2 covers their roles in other malignancies over the past five years.

Besides STs and NEUs, other molecules are also engaged in tumor sialylation. The part related to TC will be elaborated below.

## 4. Sialylation-Mediated Malignant Behaviors in TC

In the 1980s, Kokoglu *et al.* for the first time identified that TSA in serum and tissues could serve as markers for TC^22^. Later, in 2006, Pavel *et al.* demonstrated the significance of elevated sialic acid levels on the thyroid cell membrane as an important pathological indicator for distinguishing between benign and malignant thyroid lesions^29^. Other related molecular markers have also been identified to be essential in the onset and progression of PTC. Moreover, sialylation has been confirmed to be involved in the malignant progression of various subtypes of TC, including key biological behaviors such as tumor cell proliferation, dedifferentiation, migration, invasion, and immune escape. We will continue to explore the potential mechanisms of these effects (Figure 1).

### 4.1 Sialylation and Malignant Phenotypes in DTC

Tumor metastasis refers to the process by which primary tumor cells shed from the primary site and migrate to other parts of the body (e.g., nearby blood vessels or the lymphatic system, etc.) through a series of complex biological mechanisms. Beyond a defining characteristic of malignant tumors, tumor metastasis is also accepted as a major contributor to poor prognosis and mortality in cancer patients^30^. Through the alteration of their adhesion properties, sialylation of cells, the extracellular matrix, and adhesion molecules (e.g., integrins) occupy critical positions in enhancing the metastatic potential of various cancers^16^,^31^.

#### 4.1.1 Mechanisms in PTC

Abnormal regulation of sialylation can profoundly alter the glycan structures on the surface of tumor cells in PTC, thus facilitating the interactions with other cells in the tumor microenvironment (TME)^32^. It may further advance tumor invasion and metastasis. Low *et al.* discovered that in the diffuse sclerosing variant of PTC (DSVPTC), lymphoid aggregates could induce the formation of small venules resembling high endothelial venules (HEVs) found in secondary lymphoid organs. These venules further facilitated tumor cell-lymphatic endothelial cell interactions through the expression of 6-sulfo-sialyl Lewis X and intercellular adhesion molecule 1, thereby altering the TME and promoting lymph node metastasis in DSVPTC^33^. Zhang *et al.* identified a significant positive correlation between several N-glycan biomarkers and lymph node metastasis in PTC, offering potential biomarker of glycan modifications for classifying PTC patients into low- or high-risk groups for lymph node metastasis^34^. Additionally, compared to typical PTC, the follicular variant of PTC (FVPTC) had significantly higher mRNA levels of ST6GAL1, which exhibited strong correlations with lymph node metastasis, advanced clinical stage, and reduced survival^23^. Therefore, ST6GAL1 may function as a potential cancer-associated glycosyltransferase in TC, providing valuable insights into its diagnostic and prognostic significance.

#### 4.1.2 Mechanisms in FTC

FTC is the second most common form of TC, responsible for nearly 10-15% of all thyroid malignancies. Patients with FTC generally have good prognosis, despite more aggressive biological behaviors of this clinical phenotype compared to PTC^35^. Miao *et al.* discovered high expression of ST6GalNAc2 in FTC cells, which might boost tumor invasion by modulating the PI3K/Akt signaling pathway^36^. Xu *et al.* demonstrated enhanced FTC tumorigenesis after aberrant overexpression of ST6GAL2 in both *in vivo* and *in vitro* models by inhibiting the Hippo signaling pathway, which could be reversed effectively by resveratrol, a common anti-cancer compound^23^. Additionally, miR-4299 has been shown to modulate the invasive potential of human FTC cells by targeting ST6GALNAC4, both *in vivo* and *in vitro*^37^.

### 4.2 Regulation of Immune Response in DTC

Elevated levels of sialylation in tumor cells can enhance the presentation of sialylated ligands such as Mucin proteins (e.g., MUC1), CD24, and sialylated IgG on the cell surface, further facilitating their interactions with specific receptors known as Siglecs^38^-^41^. Siglecs, belonging to the immunoglobulin superfamily, are a class of glycan-binding proteins on cell surface. Their primary function is the selective recognition and binding of sialylated glycans. Through this ligand-receptor interaction, sialylated ligands on tumor cells can engage Siglecs expressed on immune cells, leading to immune tolerance, suppression of anti-tumor immunity, and facilitation of tumor progression^42^.

#### 4.2.1 Siglec-Mediated Mechanisms

To date, research has identified a total of 15 types of Siglec receptors. Tumor cells express Siglec ligands, such as CD24, which specifically bind to Siglec-10 receptors on immune cells. This interaction can transmit inhibitory signals that prevent macrophages from phagocytosing tumor cells, thereby promoting immune evasion and facilitating the malignant progression of tumors^43^. Siglecs 7, 9, 10, and 15 are generally detected to be expressed on myeloid cells, including macrophages, dendritic cells, monocytes, and neutrophils. By interacting with sialic acids, these Siglecs can interfere with the function of these immune cells, affecting cell differentiation, polarization, phagocytic ability, and immune activation, thereby contributing to immune evasion in tumors^44^. Additionally, the Siglec family may modulate the function of dendritic cells by suppressing their antigen-presenting capability, thereby weakening the initiation of specific immune responses^45^.

#### 4.2.2 Evidence from PTC Studies

In PTC cells, Maggisano *et al.* reported upregulated expression of Siglec-10 and Siglec-11 in PTC cell-derived exosomes, thereby modulating the function of immune cells^46^. Therefore, the molecular profiling of exosomes may provide valuable insights into monitoring the TME and studying the pathogenesis of TC. Huang *et al.* demonstrated the role of Siglec-15 in promoting TC cell migration by activating the PI3K/AKT signaling pathway through the epidermal growth factor receptor^47^. Jin *et al.* observed higher expression of Siglec-10 in tumor cells than that in normal tissue cells. Furthermore, co-expression of Siglec-10 and Siglec-15 in tumor cells significantly increased the risk of recurrence in PTC patients, indicating that both factors could serve as important prognostic markers and potential targets for immunotherapy in TC^48^. Additionally, by binding to Siglec-10 on macrophages, CD24 on the surface of TC cells would aid tumor cells in escaping immune surveillance^49^.

Furthermore, sialic acids were examined with higher levels on the thyroid globulin antibody IgG1 in PTC patients when compared with Graves' disease patients and healthy controls. By modulating the binding of the antibody's Fc region to Fcγ receptors, this modification of sialic acids may regulate antibody-dependent cell-mediated cytotoxicity and other immune effector functions^50^.

However, there is currently a lack of large-scale and systemic clinical data as well as animal models to elucidate the role of sialylation in the malignant progression of DTC. So far, we know little about the specific mechanisms, functions, and clinical significance of sialylation-related targets in DTC. Consequently, it is of great significance to further investigate into the expression, function, and interplay of sialylation-related molecules and immune evasion in PTC, thereby facilitating the elucidation of novel targets and strategies for immunotherapy in DTC.

### 4.3 Sialylation in Other Types of TC

Currently, researchers also focus on exploring the expression and role of sialylation in other types of TC, in addition to DTC. Preliminary studies have highlighted the potential involvement and the clinical significance of sialylation in these tumors, further supporting its value as a tumor biomarker and laying a foundation for future in-depth research.

### 4.3.1 Medullary Thyroid Carcinoma (MTC)

MTC originates from thyroid C cells, also known as parafollicular cells, which is characterized by high invasiveness, metastatic potential, and a complex genetic background^51^. So far, there is a scarcity of research on unveiling the relationship between sialylation and MTC. Specifically, Komminoth *et al.* demonstrated specific expression of poly-sialic acid in MTC, suggesting it as a potential marker for distinguishing MTC from other thyroid tumors^52^. Lewis antigens, such as sLeᵃ and sLeˣ, are a class of glycosylated antigens found on carbohydrate molecules, which are associated with the development and progression of tumor cells^53^,^54^. Vierbuchen *et al.* further revealed that sialic acids on the outermost layer of MTC tumor cells would mask the Lewis antigens, which would otherwise be recognized by the immune system, highlighting the immune-suppressive role of sialic acids in MTC^55^. This masking effect could reduce the exposure of tumor-associated carbohydrate antigens, impair immune cell recognition, and might contribute to evasion from natural killer cell-mediated cytotoxicity.

### 4.3.2 Anaplastic Thyroid Carcinoma (ATC)

Typically, ATC is highly proliferative, invasive and metastatic, which is an extremely aggressive and poorly prognostic form of TC^56^. ATC cells with elevated Siglec-15 expression exhibited a higher frequency of interactions with TME cells, further enabling tumor cells to communicate with T cells through immune-suppressive signals. Notably, blocking Siglec-15 would increase the secretion of interferon gamma (IFN-γ) and interleukin-2 (IL-2) both *in vitro* and *in vivo*, significantly inhibiting tumor growth^57^. Hou *et al.* suggested that Siglec-15 could act as a novel immune checkpoint in TC, promoting cell growth via activating the STAT1/STAT3 signaling pathway, which subsequently enhanced immune suppression. STAT1 and STAT3 are key intracellular signaling molecules that regulate cell proliferation, survival, and immune responses. In addition, silencing Siglec-15 notably inhibited TC cell proliferation and induced apoptosis, presenting a promising therapeutic approach for TC treatment^58^. Thus, high expression of Siglec-15 in ATC exhibits an intimate association with tumor invasiveness, immune evasion, and poor prognosis, providing a solid theoretical foundation for developing strategies targeting Siglec-15 for immune therapy of this disease phenotype.

## 5. The Application of Sialylation in the Diagnosis and Treatment of Thyroid Carcinoma

As previously mentioned, sialylation has been recognized to be a significant pattern of glycan modification, which has established relationship with tumorigenesis, progression, invasiveness, and metastasis. It also holds potential application value in diagnosing and treating TC, as demonstrated in the following aspects.

### 5.1 Diagnostic Applications of Sialylation

Advanced techniques, such as mass spectrometry and glycan microarrays, can facilitate the effective detection of different types of sialic acid linkages (e.g. α-2,3 or α-2,6), as well as monitoring of changes in their distribution and abundance across glycan structures^59^,^60^. These methods are invaluable for exploring the glycosylation profiles of TC tissues and their secretions, enabling precise analysis of the molecular patterns of sialylation. Existing evidence has highlighted the pivotal role of sialylation in differentiating benign from malignant thyroid nodules, and predicting lymph node metastasis^34^. As previously discussed, various STs and Siglecs are crucial molecular biomarkers for predicting TC.

Through a comprehensive analysis of plasma N-glycan alterations in patients with TC and benign thyroid nodules (BTN), Zhang *et al.* identified distinct differences in plasma N-glycomics between BTN, TC, and healthy controls. It consisted of variations in sialylation levels, and this study also constructed a prediction model, based on four potential biomarkers, to assess the risk of lymph node metastasis in TC patients^34^. Furthermore, Wallace *et al.* observed significantly lower relative abundance of N-glycans in TC tissues than that in normal thyroid tissues, potentially attributable to impaired N-glycosylation of key proteins such as thyroglobulin. Moreover, there was an escalated relative abundance of sialylated N-glycans in TC, particularly those with α-2,6 sialic acid linkages. This enrichment potentially correlated with enhanced tumor invasiveness and metastatic potential^59^. Collectively, the aforementioned findings suggest that alterations in sialylation status may serve as a valuable adjunctive diagnostic marker for TC.

### 5.2 Prognostic Implications of Sialylation

The level of sialylation has been reported to have a close link to patient prognosis. Cao *et al.* observed differences in serum N-glycomics between patients with postoperative surveillance (PS) and postoperative recurrence (PR), further confirming the indicative role of two specific glycan structures (H4N3F1L1 and H4N6F1E1) for sialylation levels in specific linkage types. These structures were found to be upregulated in patients with PR after thyroidectomy and downregulated in those without recurrence (PS), demonstrating their high predictive value for PR of TC^61^. Liu *et al.* proposed that the accumulation of sialic acids on cancer cell surfaces was a key factor influencing the morphology and invasiveness of diffuse versus non-diffuse tumor types. This hypothesis has been validated in gastric, breast, prostate, and lung cancers, which can be further applicable to other tumor types, such as DSVPTC^62^. As a result, the accumulation of sialic acids on tumor cell surfaces reveals potential correlation with tumor prognosis.

### 5.3 Therapeutic Potential of Targeting Sialylation

Great concern has been attached to the critical role of sialylation in tumor pathogenesis. Consequently, research on sialylation may both deepen our understanding of the underlying biological mechanisms of tumors, and offer potential anti-tumor therapeutic targets. Kaptan *et al.* identified that plant lectins (e.g., MAL, SNA, and AAL) might influence tumor cell survival, proliferation, migration, and interactions with endothelial cells by specifically targeting sialic acid-rich α-2,6 and α-2,3 glycan structures on the surface of TC cells^63^.

Beyond ST inhibitors, other strategies such as sialidase (neuraminidase) therapy and Siglec-targeting monoclonal antibodies (mAbs) are emerging as promising approaches. Sialidase enzymes, such as NEU1, NEU2, NEU3, and NEU4, can desialylate tumor cell surfaces, reducing hypersialylation and enhancing immune recognition^64^. For instance, in TC, sialidase treatment may reverse immune evasion by exposing tumor-associated antigens and improving T-cell-mediated cytotoxicity^65^,^66^. However, off-target effects, enzymatic stability and other similar challenges may hinder the application of sialidases^67^. Siglec mAbs, such as anti-Siglec-15 or anti-Siglec-10 antibodies, can block inhibitory signals on immune cells to restore the anti-tumor immunity. In ATC, anti-Siglec-15 mAbs have shown potential in increasing IFN-γ and IL-2 secretion to inhibit tumor growth^57^. Advantages of Siglec mAbs include high specificity and synergy with existing immunotherapies, but disadvantages are potential autoimmune reactions and the need for precise targeting to avoid immune-related adverse events^68^.

Combination therapy is emerging as a key direction in future cancer immunotherapy. Noticeably, the efficacy of immune treatments can be further enhanced by combining sialylation with other immune therapies (e.g. immune checkpoint inhibitors, CAR-T cell therapy). This approach may boost the immunogenicity of tumor cells while weakening their ability to evade immune surveillance^69^-^72^.

The necessity of targeting sialylation in TC is underscored by current treatment obstacles, such as radioiodine resistance in differentiated TC, dedifferentiation, and immune suppression in advanced cases. Sialylation-targeted strategies may contribute to overcoming these by re-sensitizing tumors to therapy, inhibiting metastasis, and reversing immune evasion. For example, in PTC, ST6GAL1 inhibition may reduce lymph node metastasis^23^, while in ATC, Siglec-15 blockade enhances immune responses^57^. However, corresponding clinical translation requires further validation due to sparse TC-specific studies.

Inhibiting sialylation, targeting sialylated glycans, and modulating immune responses to these glycans can pave the way for innovative diagnostic and therapeutic strategies, thereby improving anti-tumor therapeutic efficacy. Currently, several sialylation-related targeted therapeutic agents, ST inhibitors in particular, are under investigation. These compounds, structurally related to sialic acid donors, receptor substrates, or dual donor-receptor substrates, transition-state analogs, natural products, bile acids, and flavonoids, can specifically inhibit the sialylation of glycoproteins or glycolipids mediated by STs^73^-^75^. This blockade of the sialic acid recognition pathway can mitigate the malignant biological behaviors of tumors, such as proliferation and invasion^76^. Research on ST inhibitors has already been applied to breast cancer, melanoma, lung cancer and other malignant tumors^77^-^80^. However, given a sparse studies on TC, its clinical applications remain limited and require further investigation and validation.

## 6. Conclusions

Currently, there has been great progression in methods for detecting sialylation with the continuous advancement in biomedicine, encompassing techniques such as gas/liquid chromatography-mass spectrometry, enzyme-linked immunosorbent assays, high-performance liquid chromatography, and matrix-assisted laser desorption/ionization time-of-flight mass spectrometry^81^,^82^. Significantly, sialylation has been documented to have intimate associations with the invasiveness, metastasis, and immune regulation in various malignant tumors. However, research on the relationship between sialylation and TC is still in its infancy, with limited studies and relatively insufficient exploration. Current investigations primarily focused on alterations in sialylation expression on TC cell surfaces and its correlation with tumor biological behaviors. There is a poor understanding of the specific mechanisms, pathways, and clinical significance of sialylation in TC. For instance, we know little about the precise molecular signaling pathways and regulatory networks although sialylation has been confirmed to affect TC cell adhesion, migration, and invasiveness. Furthermore, more thorough investigation is also necessitated to unveil the role of sialylation in immune evasion in TC and its interactions with the TME. Therefore, there is a pressing need for more systematic and in-depth basic research, along with clinical validation, to explore the mechanisms of sialylation in TC and its potential value in diagnosis, treatment, and prognosis assessment. Such research may provide fresh insights and scientific evidence for precision medicine in TC, ultimately contributing to the development of more effective diagnostic tools and therapeutic strategies, thereby improving the prognosis and survival of TC patients.

## Figures and Tables

**Table 1:** Roles of related enzymes in TC

Thyroid carcinoma	Enzymes	Roles	Mechanisms
PTC	ST3GAL1	promotion	Associates with dedifferentiation^85^
	ST6GAL1	promotion	Associates with lymph node metastasis, clinical staging, and decreased survival rates^23^
	ST8SIA4	promotion	Increases metastatic potential^86^
FTC	ST6GAL2	promotion	Inactivates the Hippo signaling pathway^24^
	ST6GALNAC2	promotion	modulates the PI3K/Akt signaling pathway^36^
	ST6GALNAC4	promotion	modulates the invasion and tumorigenicity^37^

**Table 2:** Roles of STs and NEUs in malignant tumor types

Functions	Related enzymes^*^
Immune suppression or immune evasion	ST3GAL1^87^-^90^; ST3GAL3^70^; ST3GAL4^91^; ST6GAL1^92^; ST6GALNAC4^93^,^94^; ST8SIA4^95^; ST8SIA6^96^; NEU1^97^
Induction of EMT signaling pathways	ST6GALNAC1^98^; ST3GAL4^99^; ST3GAL5^100^; ST6GALNAC3^101^; NEU1^65^
Regulation of EGFR signaling	ST6GAL1^102^; ST3GAL6^103^; NEU4^104^
Regulation of immune cell infiltration	ST3GAL5^105^; ST6GAL1^106^; ST6GAL2^107^; ST8SIA1^108^
Mediation of cell-surface sialylation	ST6GAL1^109^-^113^; ST6GALNAC6^114^; ST8SIA1^115^; ST8SIA2^116^; NEU4^117^
Activation of FAK/AKT related signaling pathways	ST8SIA1^118^,^119^; ST6GALNAC1^120^
Regulation of CD8+ T cell function	ST3GAL5^121^; ST8SIA1^122^
Induction of cell death	ST6GALNAC6^123^; NEU2^124^
Promotion of the malignant biological behaviors	ST3GAL2^125^; ST6GAL1^126^,^127^; ST6GALNAC5^128^; ST8SIA4^129^; ST8SIA5^130^
Other signaling pathways	ST3GAL1: Inhibits CAR-T cells^131^; Activates the NF-κB signaling pathway^132^; ST3GAL4: Enhances glycolysis^133^; Activates MET signaling pathways^134^; ST6GAL1: Induces drug resistance^135^; Activates the cGMP/PKG pathway^136^; ST6GALNAC2: DNA repair pathway^137^; ST6GALNAC4: Activates the TGF pathway^138^; resists NK cell cytotoxicity^139^; ST8SIA1: Blocks JAK/STAT signaling pathways^140^; Enhances the Ap2α-MMP9 axis^141^ ST8SIA3: Increases A2B5 expression on the cell surface^142^; NEU3: Activates ERK and PI3K signaling^143^

^*^Literature within the latest five years

## Abbreviations

TC: thyroid carcinoma
PTC: papillary thyroid carcinoma
FTC: follicular thyroid carcinoma
DTC: differentiated thyroid carcinoma
PDTC: poorly differentiated thyroid carcinoma
ATC: anaplastic thyroid carcinoma
MTC: medullary thyroid carcinoma
TSA: total sialic acids
STs: sialyltransferases
NEUs: neuraminidases
TME: tumor microenvironment
DSVPTC: diffuse sclerosing variant of PTC
HEVs: high endothelial venules
FVPTC: follicular variant of PTC
BTN: benign thyroid nodules
PS: postoperative surveillance
PR: postoperative recurrence
sLeX: sialyl Lewis X
sLeA: sialyl Lewis A
PI3K: phosphatidylinositol 3-kinase
AKT: protein kinase B
MAPK: mitogen-activated protein kinase
Siglecs: sialic acid-binding immunoglobulin-like lectins
CMP-Neu5Ac: cytidine monophosphate-N-acetylneuraminic acid
UDP-GlcNAc: uridine diphosphate N-acetylglucosamine
ManNAc: N-acetylmannosamine
STAT1/STAT3: signal transducer and activator of transcription 1/3
IFN-γ: interferon gamma
IL-2: interleukin-2
HPLC: high-performance liquid chromatography
MALDI-TOF-MS: matrix-assisted laser desorption/ionization time-of-flight mass spectrometry
EMT: epithelial—mesenchymal transition
EGFR: epidermal growth factor receptor
CTCs: circulating tumor cells
ADM: acinar to ductal metaplasia
